# Severe rhabdomyolysis in homozygote carnitine palmitoyltransferase II deficiency

**DOI:** 10.17179/excli2020-2866

**Published:** 2020-09-11

**Authors:** Wolfgang J. Schnedl, Michael Schenk, Dietmar Enko, Harald Mangge

**Affiliations:** 1 Practice for General Internal Medicine, Dr.-Theodor-Körner-Str. 19b, 8600 Bruck/Mur, Austria; 2 Das Kinderwunsch Institut Schenk GmbH, Am Sendergrund 11, 8143 Dobl, Austria; 3 Clinical Institute of Medical and Chemical Laboratory Diagnosis, Medical University of Graz, Auenbruggerplatz 30, 8036 Graz, Austria

**Keywords:** Carnitine palmitoyltransferase II deficiency, myalgia, rhabdomyolysis, urosepsis

## Abstract

Carnitine palmitoyltransferase II (CPT II) deficiency represents an inherited defect in mitochondrial long-chain fatty acid oxidation. Rhabdomyolysis with necrosis of muscle is caused by the destruction of skeletal muscle and leads to systemic, multiorgan complications due to the release of intracellular muscle components. Severe rhabdomyolysis may be triggered by combination of a genetic predisposition, including CPT II deficiency, with additionally acting causes. Generally, patients with CPT II deficiency are rarely clinical recognized and reported. We describe a patient presenting severe rhabdomyolysis due to urosepsis, who, in genetic testing, demonstrated the homozygous CPT II deficiency (c.338C>T, p.Ser113Leu) mutation. The diagnosis of CPT II deficiency helped this patient to put the symptoms into context, and this reduced myopathy and the risk of recurring rhabdomyolysis. We report on this patient to increase awareness of diagnostic and medical management in CPT II deficiency.

## Introduction

In the inner mitochondrial membrane, carnitine palmitoyltransferase-2 (CPT-II) is involved in long-chain fatty acid (LCFA) oxidation for energy production (Houten and Wanders, 2010[5]). Inherited carnitine palmitoyltransferase II (CPT II) deficiency (c.338C>T, p.Ser113Leu) represents a defect LCFA oxidation (Joshi and Zierz, 2020[9]). Commonly, patients with CPT II deficiency are clinically not recognized (Balasubramanian et al., 2018[2]) and are therefore rarely reported (Zach et al., 2019[17]). Awareness of genetic disorders associated with rhabdomyolysis (RBD) is important to guide the diagnostic pathway. Diagnostic pathways and next-generation targeted sequencing panels allow the simultaneous analysis of several known RBD-related genes within a single assay (Scalco et al., 2015[13]). Clinical symptoms of CPT II deficiency may include no symptoms to intermittent myopathy with muscle weakness, myalgia, and rhabdomyolysis (Lehmann et al., 2017[10]). RBD is not well defined, but is regarded as destruction of skeletal muscle leading to systemic complications (Torres et al., 2015[15]). We report on this patient, who presented severe rhabdomyolysis due to urosepsis and demonstrated genetically the homozygous CPT II deficiency, c.338C>T, p.Ser113Leu mutation. However, the diagnosis of CPT II deficiency helped this patient to put the symptoms into context, and the described therapeutic measures in daily life reduced myopathy and the risk of rhabdomyolysis.

## Case Presentation

A 57-year-old male, white patient presented with stomach pain and elevated temperature with 38.2° C after an ureteroscopy. Physical examination revealed a dull, not localized, abdominal pain, muscle pain and dark, tea-colored urine. Evaluation of laboratory parameters demonstrated leukocytes 15.8 G/L (normal: 4.4-11.3), absolute neutrophils 13.7 G/L (normal: 1.8-7.7), C-reactive protein 301 mg/L (normal: <5), procalcitonin 1.7 ng/mL (normal: 0-0.5), gamma glutamyl transferase 58 U/L (normal: <55), aspartate aminotransferase 943 U/L (normal: <35), alanine aminotransferase 307 U/L (normal: <45), creatine kinase 31332 U/L (normal: <170), lactate dehydrogenase 837 U/L (normal: 120-240). Other evaluated laboratory parameters, including kidney function with creatinine and glomerular filtration rate, were within normal limits. Urine analysis with urine culture demonstrated >100.000 colony forming units per mL (CFU/mL) *Escherichia coli.* Adequate hydration with intravenous fluids and third-generation cephalosporin antibiotic therapy eased the pain, and normalized laboratory values after a week. In anamnesis, the patient reported on recurring muscle weakness and myalgia, induced mainly by strenuous exercise, fasting, and exposure to cold since childhood. With the knowledge of his, -geographically distant living-, familial CPT II deficiency the single gene testing presented a homozygous CPT II deficiency, c.338C>T, p.Ser113Leu mutation.

A registered dietician helped develop a diet with daily frequent meals, to avoid prolonged fasting, with daily sufficient hydration, and with the suggestion of high-carbohydrate (70 %) and low-fat (<20 %) meals. Advice was provided to circumvent strenuous exercise, medications including ibuprofen, valproic acid, statins, diazepam, and to avoid general anesthesia-related hazards. Available vaccinations for infectious diseases, including the annual flu, were recommended. After one year the patient presented with rare intermittent, mild myalgia, and laboratory values, including serum creatine kinase (CK) concentrations, within the normal range. Written informed consent was obtained for all procedures, which were in accordance with the Declaration of Helsinki and the recommendations of the local ethics committee.

## Discussion

Glucose, fatty acids and amino acids are the basis for human energy production. Approximately 95 % of total body carnitine is found in skeletal muscle, as carnitine is essential while exercising muscle for fatty acid oxidation. Some carnitine is also in the liver, heart, and kidneys. Carnitine operates through acetylcarnitin, which enters the energy-producing citric acid cycle (Krebs's cycle). Inherited disorders may involve carnitine biosynthesis, membrane transport deficiencies and defects in mitochondrial carnitine-acylcarnitine cycle (Almannai et al., 2019[1]). 

However, the mitochondrial membrane is impermeable to long-chain fatty acids (LCFAs) and this requires a LCFA transport system with several transport steps, namely the carnitine shuttle. Degradation of plasma free fatty acids or lipoprotein-associated triglycerides is mainly seen within mitochondrial fatty acid β-oxidation of LCFAs. The enzyme carnitine palmitoyltransferase (CPT) is involved in this transfer of LCFAs from cytoplasm into mitochondria. In the outer mitochondrial membrane carnitine palmitoyltransferase-1 (CPT-I) converts an acyl-CoA into acylcarnitine, which subsequently passes through the membrane (Houten and Wanders, 2010[5]). 

CPT I deficiency, for which isolated patients very rarely, have been characterized, seems a disease predominantly of hepatic mitochondrial LCFA oxidation. It is described as hepatic and cardiac failure causing early death in newborns (Janeiro et al., 2019[7]). Nonetheless, CPT I deficiency was also described in an adult, recently (Phowthongkum et al., 2019[12]). 

In the inner mitochondrial membrane, carnitine palmitoyltransferase-2 (CPT-II) converts acylcarnitine back to long-chain fatty acyl-CoAs (LCFA-CoAs) and carnitine for energy production. In CPT II deficiency, the conversion of acylcarnitine into LCFA-CoAs is insufficient and only parts of acylcarnitine diffuse across the inner mitochondrial membrane (Houten and Wanders, 2010[5]). This explains an increase of acylcarnitines (C12 to C18) in the plasma of affected patients. However, acylcarnitine determination in plasma may help to diagnose a CPT II deficiency (Wieser, 2019[16]). 

CPT II deficiency is inherited, autosomal recessive, and reported with three phenotypes. Two disorders represent severe multi-systemic diseases with a lethal neonatal and a severe infantile hepato-cardiomuscular form. Both are characterized by liver failure with hypoketotic hypoglycemia, cardiomyopathy, seizures, and early death (Wieser, 2019[16]). 

However, the most frequent type of CPT II deficiency, c.338C>T, p.Ser113Leu, occurs with mild myopathy and an approximate frequency of more than 60 %. This form of CPT II deficiency may be found at any age, but approximately 70 % appear early during childhood. Several other genetic variants are associated with mild and/or severe forms of myopathy (Joshi and Zierz, 2020[9]). Additional pathogenetic, some not yet known, mechanisms are discussed. Symptoms are mainly induced by exercise or exposure to cold with muscle pain and weakness, sometimes with myoglobinuria (Scalco et al., 2015[13]; Torres et al., 2015[15]; Wieser, 2019[16]). Although, heterozygote carriers are generally asymptomatic, myopathy in heterozygote CPT II patients has also been reported (Joshi et al, 2012[8]).

Biochemical consequences of various disease-causing mutations are controversial and still being discussed (Lehmann et al., 2017[10]). Bezafibrate, as a triglyceride lowering medication, and supplements with L-carnitine are being considered as supportive in treatment of CPT II deficiency (Olpin et al., 2015[11]). While our patient opposed any medication he also demonstrated only intermittent, mild myalgia after following the suggestions indicated above. 

Medical personnel was not aware of the CPT II deficiency and therefore, single-gene (Sanger) sequencing was performed in the recovered patient, after he presented his family investigations (Zach et al., 2019[17]). Awareness of supposed genotype-phenotype correlations in various genetic disorders associated with RBD is important to guide the diagnostic pathway (Scalco et al., 2015[13]). Diagnostic algorithms and next-generation targeted sequencing using a RBD-targeted gene panel allow simultaneous analysis of several known RBD-related genes (Olpin et al., 2015[11]). Following suggested diagnostic pathways seems that genetic testing for muscle disorders in clinically recognized patients will improve medical management.

RBD is characterized by destruction of skeletal, striated muscle, with release of intracellular muscle content, leading to multiorgan complications. Generally, RBD is inhomogeneous and not well defined. Bacterial infections may cause RBD (Deng et al., 2017[4]). Urosepsis caused by ureteroscopy is considered a potentially life-threatening infection (Scotland and Lange, 2018[14]). RBD may be caused by several different reasons (Table 1(Tab. 1); References in Table 1: Balasubramanian et al., 2018[2]; Lehmann et al., 2017[10]; Houten and Wanders, 2010[5]). The interpretation of laboratory parameters in RBD varies among studies, defining a severe RBD with CK values from >5.000 up to >15.000 U/L. Excessive RBD appears by combination of a genetic predisposition with additionally acting triggers. 

The clinical presentation of RBD ranges from muscle pain and weakness, pigmenturia with dark, tea-colored urine, to vomiting and confusion. Acute kidney failure is the most common complication and occurs with a frequency of up to 55 percent. Generally, RBD is mainly associated with a poor prognosis when combined with multiorgan failure. So far, guidelines for the medical management of RBD are nonexistent (Chavez et al., 2016[3]). The mainstay of treatment stays the preservation of renal function with adequate intravenous fluid therapy (Ivin et al., 2020[6]). It is important to stay alert for the clinical syndrome of RBD and react appropriately to prevent serious complications with kidney failure or even death. 

So far, inherited, genetic muscle disorders associated with RBD are heterogeneous. Between symptoms, most patients with CPT II deficiency, c.338C>T, p.Ser113Leu, have normal serum CK concentrations. Only 10 % of patients show permanent elevation of serum CK values. A considerable amount of patients presenting with RBD remain genetically unexplained. CPT II deficiency appears to be more common than anticipated, but it is not sufficiently recognized and, therefore, rarely reported (Balasubramanian et al, 2018[2]; Zach et al., 2019[17]). Polymorphisms causing an additive effect in the pathogenesis of RBD have been suggested, but they proved difficult to be confirmed (Scalco et al., 2015[13]). 

Overall, we report on this patient to raise awareness and increase understanding of medical management in CPT II deficiency. When patients repeatedly present with myalgia, triggered only by minor causes, a thorough work-up for a possible underlying myopathy, including genetic testing (Scalco et al., 2015[13]; Olpin et al., 2015[11]), should be initiated. The diagnosis of CPT II deficiency, c.338C>T, p.Ser113Leu, helped this patient to put the symptoms into context and this enabled him to adapt in everyday-life, which thereby reduced myopathy and the risk of recurring RBD.

## Funding

The authors have received no funding for this manuscript.

## Conflict of interest

All authors declare no conflict of interest.

## Figures and Tables

**Table 1:**
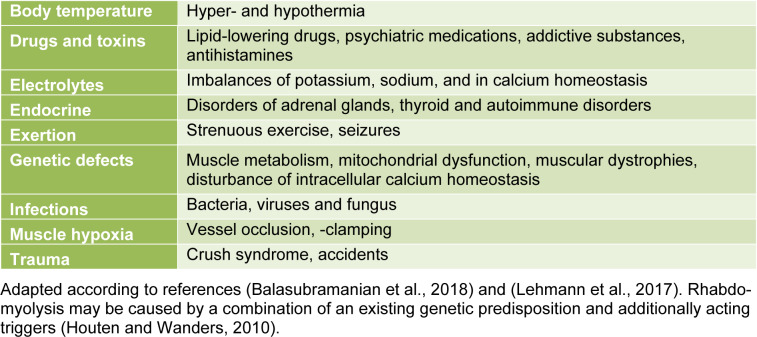
Examples for triggers of rhabdomyolysis
